# What do Differences in Emotional Regulation in Individuals Addicted to Different Substances Tell us About Addiction Treatment?

**DOI:** 10.5812/ijhrba.14630

**Published:** 2013-09-20

**Authors:** Frank Scott Hall

**Affiliations:** 1 Molecular Neurobiology Branch, NIDA-IRP/NIH/DHHS, Baltimore, USA

**Keywords:** Suppression, Reappraisal, Opioid, Methamphetamine

*Dear Editor,*

Despite the high incidence of drug abuse, dependence and addiction worldwide, treatments for these conditions remain minimally effective. Among the reasons that might contribute to the limited efficacy of both pharmacological and behavioral/psychological treatments for addictions are symptomatic and etiologic heterogeneity. Although some addicts are predisposed to addiction toward multiple substances, many others appear to be more selectively disposed towards particular substances. This may reflect, to some degree, cultural biases that are unrelated to biological or psychological factors, resulting in limited access to certain drugs (such as alcohol in predominantly Moslem countries) or other aspects of drug availability, such as cost related to where drugs are produced. On the other hand, these differences may involve individual differences in psychological attributes that contribute to addiction and successful quitting. In a recent article in International Journal of High Risk Behaviors and Addiction by H. Mohajerin et al. (1) several interesting aspects of differences between individuals addicted to drugs of specific classes were examined. This study has implications for both the causes of addiction and its treatment.

Mohajerin et al. examined styles of emotional regulation in individuals addicted to opiates or amphetamines. The idea behind this study was that differences in emotional regulation might differ in individuals addicted to different drugs. Using the Emotion Regulation Questionnaire (2), differences in emotional regulation style were examined in opiate-dependent and methamphetamine-dependent subjects. The results revealed that opiate-dependent subjects had higher scores on the emotional suppression scale, while methamphetamine-dependent subjects had higher scores on the reappraisal scale. The authors suggested that this may relate to differences in other psychiatric comorbidities among the subjects, including depression in opioid-dependent subjects. There are differences in psychiatric comorbidities in individuals based on different drug types, although this may more reflect the presentation of the disorder (3, 4).

Mohajerin et al. emphasized types of cognitive-behavioral interventions involving active restructuring of interpretations of negative experiences for methamphetamine users, who tended to have this way of thinking to begin with. Although interesting, it does not necessarily follow that therapeutic approaches should follow such tendencies. Perhaps, despite the fact that opiate-dependent individuals have a tendency away from cognitive reappraisal towards suppressive strategies in emotional regulation, therapy should aim at ameliorating this weakness and improving cognitive reappraisal abilities. Two empirical questions arise which require further study: “Do different therapeutic approaches work better in individuals addicted to particular circumstances?”, and “Are such differences primarily premorbid in origin or partially the result of chronic drug exposure?” 

This “which came first” question becomes especially important in considering the etiology of addiction to opiates and stimulants. A substantial portion of the propensity to develop addiction is heritable and involves a large number of genes. However, whether this genetic basis is primarily polygenic or highly heterogeneous is a matter of some debate (5, 6). Based on genome-wide association studies, classes of genes other than drug targets appear to be primarily involved in addiction (6). This genetic variation could still alter sensitivity to addictive drugs in a less direct fashion, but these genes may also affect behavioral or psychological phenotypes such as those considered by Mohajerin et al. As Mohajerin et al. suggest, some portion of this predisposition might relate to attempts at self-treatment for co-morbid psychiatric conditions. Other genetically mediated behavioral or psychological characteristics might predispose individuals to addiction by affecting cognitive or behavioral traits that influence drug-taking behavior, such as impulsivity. Finally, an overlapping, but not entirely the same, set of genes may contribute to the ability to quit drugs or to the success of particular types of behavioral/psychological or pharmacological therapies, as initial studies of nicotine cessation have suggested (7, 8).
